# Supplementary material for: Structural insights into ribosomal rescue by Dom34 and Hbs1 at near-atomic resolution
Source: Nat Commun. 2016 Dec 20;7:13521. doi: 10.1038/ncomms13521 (PMC5187420; doi:10.1038/ncomms13521)
Supplement: Supplementary Information — Supplementary Figures 1-5 and Supplementary Table 1. [file ncomms13521-s1.pdf]

Supplementary Material

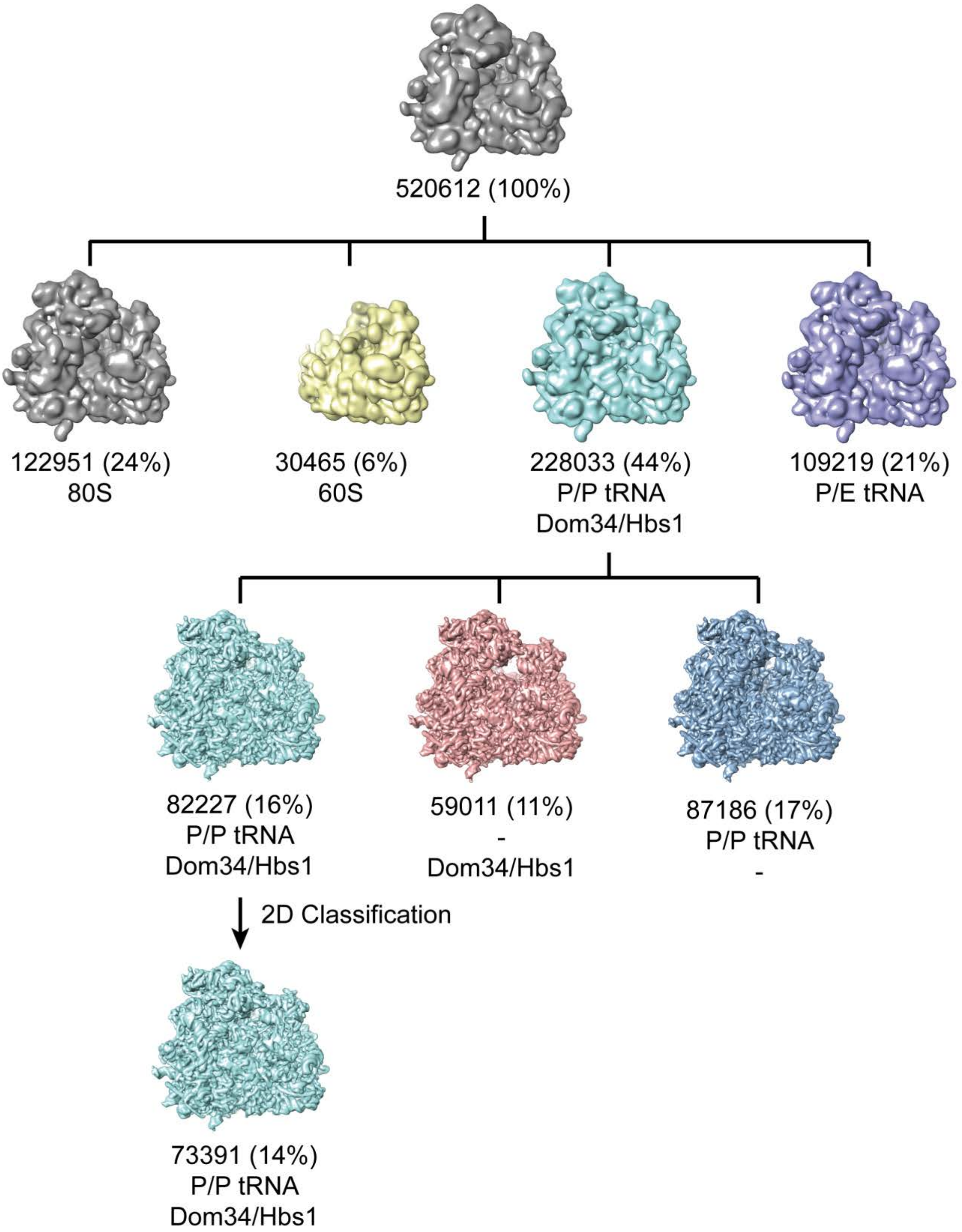

Supplementary Figure 1: *In silico* sorting scheme of the nonstop ribosomal complex. After several iterations of refinement with SPIDER a homogeneous population of 82,227 particles was obtained containing all desired components. Subsequently, RELION was used for final clearance of the dataset by 2D classification.

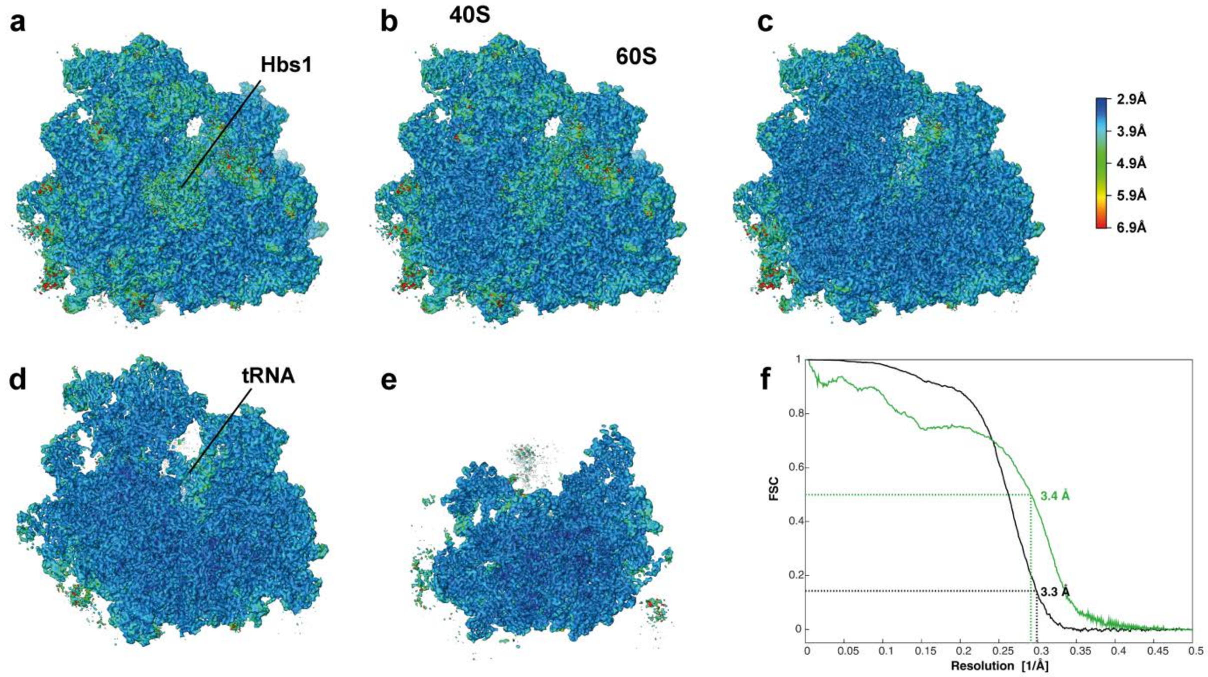

Supplementary Figure 2: resolution measures of the nonstop ribosomal complex. (a-e) represent slices through the density map, colored by the local resolution estimated with ResMap. 40S and 60S subunits are resolved equally well, with a resolution better than 3Å in the core. Flexible regions, especially at the periphery are less well resolved. **(f)** overall resolution determined by the FSC of two half-maps (black) or by cross-correlation of the refined atomic model with the density map (green).

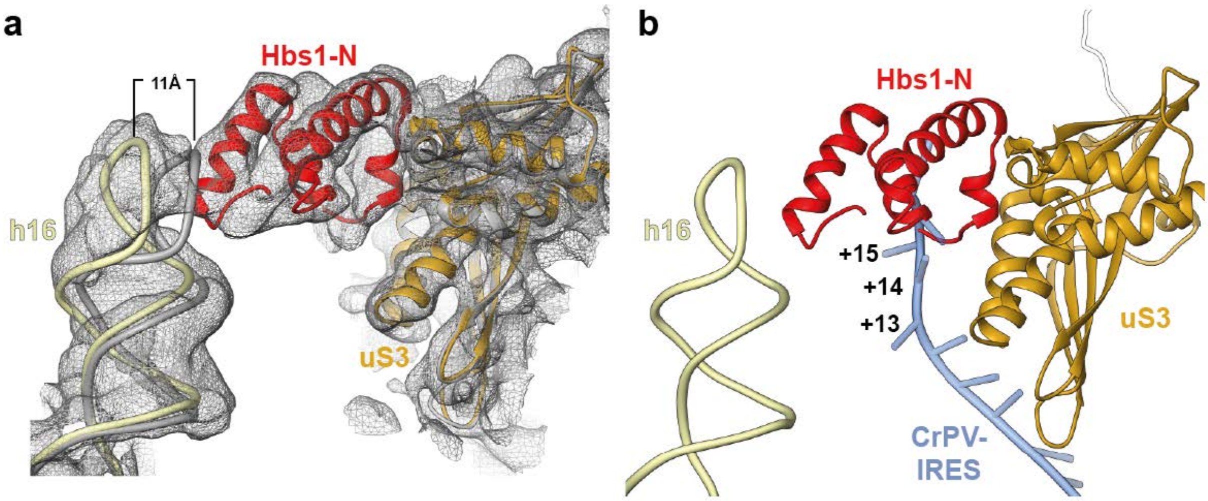

Supplementary Figure 3: The N-terminal domain of Hbs1 helps substrate recognition. **(a)** The electron density map surrounding the mRNA entry channel in the nonstop ribosomal complex (NsRC) is shown filtered to 5 Å. Hbs1-N (red) is placed between h16 (yellow) and uS3 (gold). The tip of h16 is displaced by 11 Å compared to the position in the vacant ribosome (grey). (**b**) Superposition of the CrPV-IRES (blue) from the related ribosomal termination complex[1] into our NsRC illustrates that the binding position of Hbs1-N overlaps with downstream nucleotides of long mRNAs. It seems likely that Hbs1-N competes with mRNAs extending the P-site for binding on the mRNA entry site starting with the nucleotides in position +14.

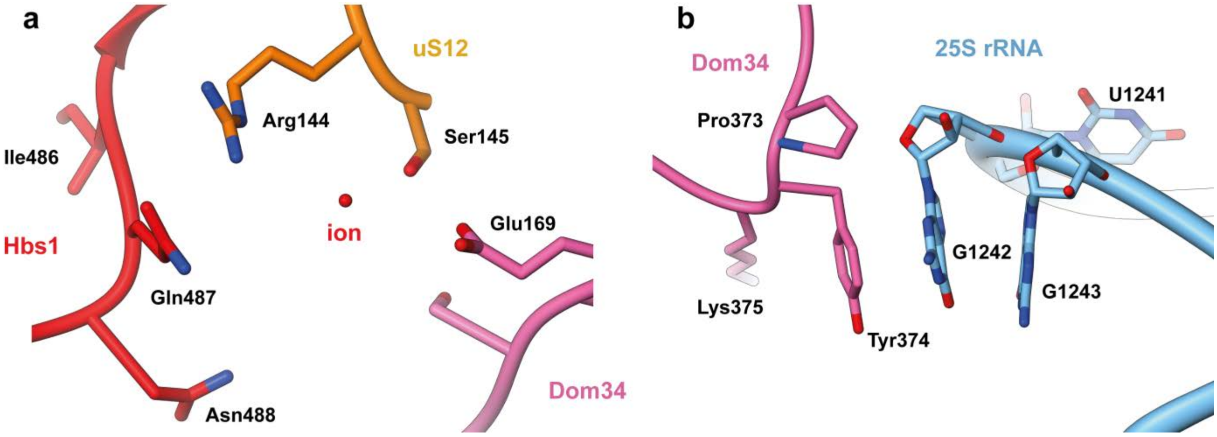

Supplementary Figure 4: Selected interactions of the rescue factors with the ribosome
(a) Gln487 of Hbs1 (red) and Glu169 of Dom34 (hot pink) are not in contacting distance. Arg144/Ser145 of the ribosomal protein uS12 (orange) are in proximity and mediate the interaction between the rescue factors via an coordinated ion. (**b)** Tyr374 of Dom34 (hot pink) is stacking on G1242 of the 25S rRNA (blue) forming a close contact to the ribosomal 60S subunit.

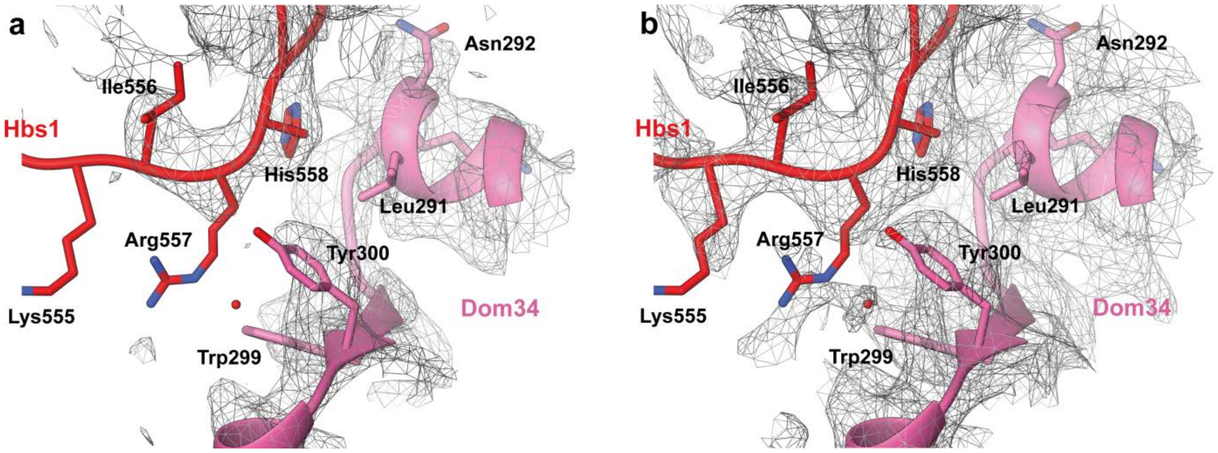

Supplementary Figure 5: Surrounding of the conserved Tyr300 at different contour levels (a) Tyr300 of Dom34 (hot pink) is pointing towards Hbs1 (red). The displayed contour level is 3. **(b)** Contour level of 2. Density for some side chains becomes more visible, e.g. Lys555 of Hbs1.

Supplementary Table 1: Data collection, refinement and validation

**Data collection**	
Particles	73391
Pixel size (Å)	0.994
Defocus range (μm)	-0.5-4.5
Voltage (kV)	300
Electron dose (e^{-}/$Å^{-2}$)	25
Resolution (Å, FSC 0.143)	3.3
**Model composition**	
Protein residues	13128
RNA residues	5444
**Model refinement**	
Applied weight	1
**Protein**	
RMS deviation	
Bond lengths (Å)	0.01
Bond angles (°)	0.07
Ramachandran favored (%)	93
Ramachandran outliers (%)	0.13
Rotamer outliers (%)	0.01
**RNA**	
Correct sugar puckers (%)	98.6
Good backbone conformations (%)	82.4
**Clashscore**	**3.7**
